# Impact of modulating leptin sensitivity on the transcriptomic profile of adult-derived hypothalamic mouse neurons

**DOI:** 10.3389/fnmol.2024.1518737

**Published:** 2025-01-17

**Authors:** Ewa Ocłoń, Artur Gurgul, Tomasz Szmatoła, Igor Jasielczuk, Miroslaw Kucharski, Joanna Zubel-Łojek, Dorota Anna Zieba

**Affiliations:** ^1^Laboratory of Recombinant Proteins Production, University of Agriculture in Krakow, Krakow, Poland; ^2^Department of Basic Sciences, University of Agriculture in Krakow, Krakow, Poland; ^3^Department of Animal Physiology and Endocrinology, University of Agriculture in Krakow, Krakow, Poland; ^4^Department of Animal Biotechnology, University of Agriculture in Krakow, Krakow, Poland

**Keywords:** leptin, hypothalamic neurons, palmitic acid, leptin resistance, inflammation, RNA-seq and Traf5, the C-type lectin receptor signaling pathway (Nfkb2), IRF9

## Abstract

The modulation of leptin sensitivity in hypothalamic neurons plays a crucial role in metabolic regulation and the development of obesity. Three distinct approaches, exposure to leptin (LEPA), administration of leptin antagonist (LANTA), and treatment with palmitate (PA), were explored in this study to assess their effects on adult-derived mHypoA-2/12 neurons and the resulting transcriptomic signatures. To this end, 3’ mRNA-Seq transcriptome analysis was employed, unexpectedly revealing downregulation of key genes associated with the NOD-like receptor signaling pathway (*Irf9*, *Mapk3*, *Stat2*, *Nfkbia*, *Ikbkg*, *Rela*, *Cxcl1*, and *Traf5*), the C-type lectin receptor signaling pathway (*Nfkb2*, *Irf9*, *Mapk3*, *Stat2*, *Nfkbia*, *Ikbkg*, *Rela*, and *Ptgs2*), the NF kappa B signaling pathway (*Nfkbia*, *Ikbkg*, *Nfkb2*, *Rela*, *Traf5*, *Cxcl1*, and *Ptgs2*), and the IL 17 signaling pathway (*Nfkbia*, *Ikbkg*, *Mapk3*, *Rela*, *Traf5*, *Cxcl1*, and *Ptgs2*). These findings help elucidate the molecular mechanisms through which these factors influence leptin sensitivity and provide insights into the pathways implicated in the development of leptin resistance in hypothalamic neurons. The surprising downregulation of these pathways suggests a complex interplay between leptin signaling and the cellular stress response in hypothalamic neurons. This alteration may reflect adaptive mechanisms in response to prolonged leptin or fatty acid exposure. Understanding these dynamics is essential for elucidating the role of hypothalamic inflammation in the progression of leptin resistance and associated metabolic disorders.

## 1 Introduction

Hypothalamic leptin resistance, a critical factor in the development of obesity and related metabolic disorders, is driven by various mechanisms, including hyperleptinemia, inflammation, endoplasmic reticulum (ER) stress, and defective autophagy. Hyperleptinemia, characterized by chronically elevated leptin concentrations, causes leptin resistance by overactivating negative feedback regulators, ultimately impairing the leptin signaling pathway (Zieba et al., 2008, 2020; Szczesna et al., 2011). Neuroinflammation, particularly in the hypothalamus, is an important factor in leptin resistance. The hypothalamus, a key regulator of food intake and energy expenditure (Williams, 2012), is the focal point of our search for the causes of leptin resistance of various origins (Zieba et al., 2019, 2023; Biernat et al., 2021). High-fat diets (HFDs) induce hypothalamic inflammation through the activation of toll-like receptor 4 (TLR4), leading to impaired leptin signaling (Chen et al., 2024). The accumulation of misfolded proteins in the ER triggers stress responses that can disrupt leptin signaling. This mechanism is particularly evident in obesity, where ER stress in the hypothalamus exacerbates leptin resistance (Zhou and Rui, 2013; Horiuchi et al., 2021). Finally, defective autophagy, a crucial process in cellular homeostasis, has been linked to leptin resistance. Dysregulated autophagy in hypothalamic neurons impairs energy balance and contributes to obesity (Kamareddine et al., 2021). Each of these mechanisms underscores the complex interplay between metabolic dysfunction and leptin signaling, highlighting the challenges in managing leptin resistance and obesity.

To modulate leptin sensitivity, various strategies, including the application of leptin, palmitate (PA), or leptin antagonists (LANTAs), have been employed in *in vitro* models; each approach affects leptin signaling through distinct mechanisms. Prolonged exposure of cells to leptin can lead to overstimulation of the JAK2-STAT3 signaling pathway. This overstimulation results in the upregulation of suppressor of cytokine signaling 3 (SOCS3), a key negative feedback regulator that diminishes leptin receptor signaling, ultimately reducing cellular sensitivity to leptin (Liu et al., 2022). This approach closely models leptin resistance in obesity, in which chronically high leptin concentrations contribute to a diminished response to the hormone.

On the other hand, PA, the primary saturated fatty acid in the Western diet and a significant contributor to human obesity, induces leptin resistance through mechanisms involving ER stress and inflammation. Exposure to PA activates inflammatory pathways, such as the NF-kappa B pathway, and promotes the expression of SOCS3, thereby disrupting leptin receptor function and impairing leptin signaling (Chen et al., 2024). This model is particularly useful for studying how dietary factors contribute to metabolic disorders and the development of leptin resistance.

LANTAs modulate leptin sensitivity by directly blocking the leptin receptor. By preventing leptin from binding to its receptor, these antagonists effectively inhibit downstream signaling, creating a state of artificial leptin resistance. This approach not only helps us explore the physiological roles of leptin but also has potential value in the development of therapeutic strategies for obesity and related conditions. This approach is a step toward a future in the fight against metabolic disorders, offering hope that effective treatments can be developed (Philp et al., 2021).

Although these methods effectively reduce leptin sensitivity, they all employ different mechanisms: leptin-induced resistance arises from receptor overactivation, PA-induced resistance from lipid-induced stress and inflammation, and LANTA-induced resistance from direct receptor blockade. Understanding these distinctions is crucial for developing targeted therapies to counteract leptin resistance and its metabolic consequences.

In this study, the effects of three distinct factors—leptin (LEPA), leptin antagonists (LANTAs), and palmitate (PA)—on adult-derived mHypoA neurons were investigated, and the resulting transcriptomic signatures were analyzed, providing highly valuable results. The aim of this study was to elucidate the molecular mechanisms by which these factors modulate leptin sensitivity and identify the genetic pathways implicated in the development of leptin resistance. This study provides invaluable insights into the complex world of leptin resistance and its modulation, thereby contributing to endocrinology, metabolism, and obesity.

## 2 Materials and methods

The Second Local Ethics Committee on Animal Testing in Krakow, Poland, approved the experiments (Protocols No. 95/2024). The adult-derived mHypoA-2/12 cell line is an appropriate line for investigating PA/leptin effects, neuroinflammation, and leptin resistance in hypothalamic neurons.

### 2.1 Cell culture and treatments

The adult mouse hypothalamic cell line mHypoA-2/12 (Clu177, Cedarlane, Canada) was grown in Dulbecco’s modified Eagle’s medium (DMEM) with GlutaMAX (4,500 mg/L, Gibco - Thermo Fisher Scientific, United States) supplemented with 10% heat-inactivated fetal bovine serum (FBS, Gibco, Thermo Fisher Scientific, United States) and 1% Gibco™ Antibiotic-Antimycotic (10,000 units/mL penicillin, 10,000 μg/mL streptomycin, and 25 μg/mL Gibco Amphotericin B, Thermo Fisher Scientific, United States). Briefly, the cells were plated in 24-well culture plates at a density of 1.5 × 10^5^ cells/well and maintained at 37°C and 5% CO_2_. The cells were exposed to mouse leptin (LEPA: 200 nM, PLR, Israel), mouse leptin antagonist (LANTA: 30 μM, PLR, Israel), or palmitic acid (PA, 5 mM, Merck, United States) for 24 h. PA was conjugated with fatty acid-free bovine serum albumin (NEFA-free BSA) and reconstituted in serum-free DMEM (Thermo Fisher Scientific, United States) as previously described (Mayer and Belsham, 2010). Vehicle controls were cultured in serum-free DMEM containing 5% NEFA-free BSA (CTR). After treatment, the cells were used for the assays described below.

### 2.2 Cell viability assay

Cell viability was assessed using the RealTime-Glo™ MT Cell Viability Assay (Promega, United States) according to the manufacturer’s protocol. The treated cells were reseeded in white-walled 96-well plates at a density of 4,000 cells/well and allowed to attach overnight. For time zero measurements, the cells were incubated with RealTime-Glo™ MT Cell Viability reagent for 20 min at 37°C, and the luminescence intensity was measured with a TECAN Infinite M200 PRO microplate reader. The data are reported as the relative luminescence compared with the control.

### 2.3 Caspase-3/7 activity assay

Caspase-3/7 activity was measured using the Caspase-Glo® 3/7 Assay (Promega, United States). Briefly, mHypoA-2/12 cells (15 × 10^3^ cells per well) were cultured in a 96-well white plate. For the positive control, the cells were treated with 5 μM staurosporine (0.1% final DMSO, Merck, United States) for 24 h. Caspase-Glo® 3/7 reagent was added to all the wells at a 1:1 ratio, and the plate was shaken at room temperature for 30 min. The luminescence was measured, and the data were reported as described previously.

### 2.4 Calcium fluorescence assay

Intracellular calcium levels were measured using a Fluo-4 NW calcium assay kit (Invitrogen) according to the manufacturer’s instructions. Treated cells were seeded into black-walled, clear-bottom 96-well plates at a density of 10,000 cells/well and allowed to attach overnight. After incubation with Fluo-4 NW dye in the presence of 2.5 mM probenecid for 30 min at 37°C, the fluorescence intensity was measured with a TECAN Infinite M200 PRO microplate reader with excitation at 494 nm and emission at 516 nm. The data are reported as relative fluorescence units (RFUs) compared with the control.

### 2.5 3’ mRNA-Seq transcriptome analysis

Treated and control cells were trypsinized, transferred to 1.5 mL Eppendorf-type tubes, and centrifuged at 500 × g for 7 min. The supernatants were immediately removed, and the pelleted cells were snap-frozen at −80°C until RNA isolation. TRI Reagent™ Solution (Thermo Fisher Scientific, United States) was used to purify total RNA according to a standard procedure. The quality of the RNA was assessed with the TapeStation 4,150 System (Agilent, United States). RNA quantification was performed using the Qubit RNA BR assay (Thermo Fisher Scientific, United States). The library was prepared from 50 ng of total RNA with the QuantSeq 3’ mRNA-Seq Library Prep Kit FWD (Lexogen, Austria), which enables the performance of standard gene expression analysis with as few as 3 million (M) sequencing reads per sample. The quality of the prepared indexed libraries was checked with the TapeStation 4,150 System (Agilent, United States) and D1000 ScreenTape. Libraries were quantified using a Qubit dsDNA BR kit (Thermo Fisher Scientific, United States). The libraries were pooled in equimolar concentrations and finally sequenced in a single-end 150 bp run on a NovaSeq 6,000 System (Illumina, United States) to obtain at least 6 M reads per library. Raw sequencing reads and raw read counts were publicly shared in the Gene Expression Omnibus (GEO) and Sequence Read Archive (SRA) databases held by the National Center for Biotechnology Information (NCBI) under the accession number GSE280030.

### 2.6 Sequencing data analysis

FastQC (v0.11.9) software was used to evaluate the quality of the raw sequencing reads. Then, trimming and filtering were performed using Flexbar software (3.5.0) (Dodt et al., 2012). During filtering, quality end reads, adapter sequences, and reads that were too short after trimming were removed. The clean, high-quality reads were mapped to the mouse GRC39 assembly with the STAR aligner (2.7.5c) (Dobin et al., 2013). For read counting within annotation MM109 (Ensembl) features, Htseq-count (1.99.2) (Anders et al., 2015) software was used. Final read normalization, principal component analysis (PCA), hierarchical clustering, and differential expression (DE) analysis via DESeq2 (v3.16) software (Love et al., 2014) were performed on the iDEP.96 (v1.1) server (Ge et al., 2018). The function and enrichment in specific Gene Ontology (GO) terms [biological process (BP)] and Kyoto Encyclopedia of Genes and Genomes (KEGG) pathway categories in differentially expressed genes [false discovery rate (FDR) < 0.05] were analyzed via iDEP.96 software. The analyzed BP terms and KEGG annotation categories were considered enriched when the corresponding FDR was lower than 0.05.

### 2.7 Statistical analysis

Statistical analyses were conducted using JASP v0.17.1 software.^1^ The distribution of the data was first assessed via the Shapiro–Wilk test, and variance equality was examined via the Levene test. For data with a normal distribution and equal variances, one-way ANOVA was performed, followed by the Tukey *post hoc* test for group comparisons. When the data did not follow a normal distribution or showed significant differences in variance, the nonparametric Kruskal–Wallis test was used as an alternative to ANOVA, with group comparisons analyzed via the Dunn post hoc test. For validation of RNA-seq results, Pearson correlation coefficients were calculated to compare gene expression levels obtained through RNA-seq and qPCR. A strong positive correlation between the two methods (*r* > 0.85, *p* < 0.05) confirmed the reliability of the transcriptomic data.

### 2.8 qPCR validation of the transcriptome data

Quantitative PCR (qPCR) analysis of three selected genes (*Mapk3*, *Stat2* and *Stat3*) was performed using the TaqMan Gene Expression Assay (Thermo Fisher Scientific) on a StepOnePlus™ Real-Time PCR System (Applied Biosystems; Thermo Fisher Scientific). Total RNA was isolated from three biological replicates for each experimental condition (Control, PA, LEPA, and LANTA). For each sample, 500 ng of RNA was reverse transcribed into complementary DNA (cDNA) using the High-Capacity RNA-to-cDNA Kit (Thermo Fisher Scientific) according to the manufacturer’s protocol. The resulting cDNA was used as a template for qPCR.

Each sample was analyzed in triplicate under the following PCR conditions: 50°C for 5 min; 95°C for 10 min; and 40 cycles at 95°C for 15 s and 60°C for 1 min. The relative expression levels of each gene were calculated via the ΔΔCt method, with gene expression levels standardized to those of *GAPDH* and 18S rRNA as the endogenous controls.

## 3 Results

### 3.1 Assessment of cell viability

**Figure 1:**
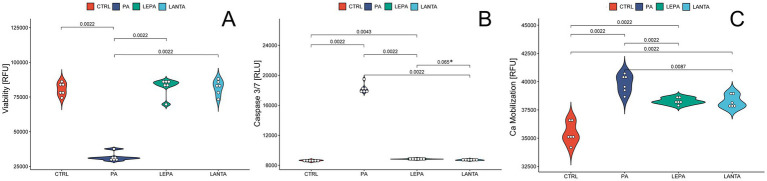
Effects of palmitate (PA), leptin (LEPA), and leptin antagonist (LANTA) on key cellular functions in hypothalamic neurons. The graph shows cell viability **(A)**, Caspase 3/7 activity **(B)** and Calcium mobilization **(C)** altered by PA, LEPA, and LANTA treatments compared with the control (CTRL).

To evaluate cell viability, we performed the RealTime-Glo™ MT Cell Viability Assay (Promega). A 24-h incubation period with LEPA (200 nM) or LANTA (30 μM) maintained cell viability at levels comparable to those of the control (Figure 1A). In contrast, incubation with PA (5 mM) for 24 h significantly decreased cell survival by 60.75% (*p* < 0.001).

### 3.2 Assessment of caspase-3/7 activity

To quantitatively assess apoptotic activity, we performed the Caspase-Glo® 3/7 Assay (Promega), which specifically measures the activity of caspase-3 and caspase-7, which are central executors of apoptosis. After a 24-h incubation period, LEPA (200 nM) and LANTA (30 μM) did not significantly alter caspase-3/7 activity compared with that in the control group, indicating that these treatments did not trigger significant apoptotic responses. In contrast, treatment with PA (5 mM) resulted in a marked increase in caspase-3/7 activity, with a fold change of 2.12 relative to that of the control (*p* < 0.001), confirming the induction of apoptosis by PA (Figure 1B).

### 3.3 Calcium assay

To investigate the effects of LEPA, LANTA, and PA on intracellular calcium levels, we used the Fluo-4 NW Calcium Assay Kit (Invitrogen). Following a 24-h incubation period, significant increases in intracellular calcium levels were observed in all the treatment groups compared with those of the control group. Specifically, treatment with PA (5 mM) resulted in a substantial increase in calcium levels, with mean RFU values increasing from 35,448.83 (control) to 39,830.67 (*p* < 0.01). Similarly, LEPA (200 nM) and LANTA (30 μM) also induced significant increases in calcium levels, with mean RFU values increasing to 38,282.67 (*p* < 0.05) and 38,246.83 (*p* < 0.05), respectively (Figure 1C). While PA elicited a more pronounced calcium response compared with the control than the other two treatments did, the differences among PA, LEPA, and LANTA were not statistically significant, indicating that all three treatments effectively elevated the intracellular calcium levels to a similar extent.

### 3.4 Sequencing data evaluation and general transcriptome profile differentiation

Overall, 3’ mRNA-Seq sequencing reads were generated for 15 cell cultures classified into four treatment groups (CTR, PA, LEPA, and LANTA). On average, 6.5 M (SD = 693.9 K) raw reads per sample were generated, of which 6.37 M (SD = 668.0; 97%) passed initial filtering. Among the filtered reads, on average, 3.8 M (SD = 426.6; 60.4%) were uniquely mapped to the reference genome assembly. A mean of 3.7 M (SD = 417.6; 97.5%) reads were counted in genes and used for differential expression analysis (Supplementary Table S1). Among the initial 57,010 annotated genes, 27,978 passed the expression level filter and were used in the final differential expression analysis.

**Figure 2:**
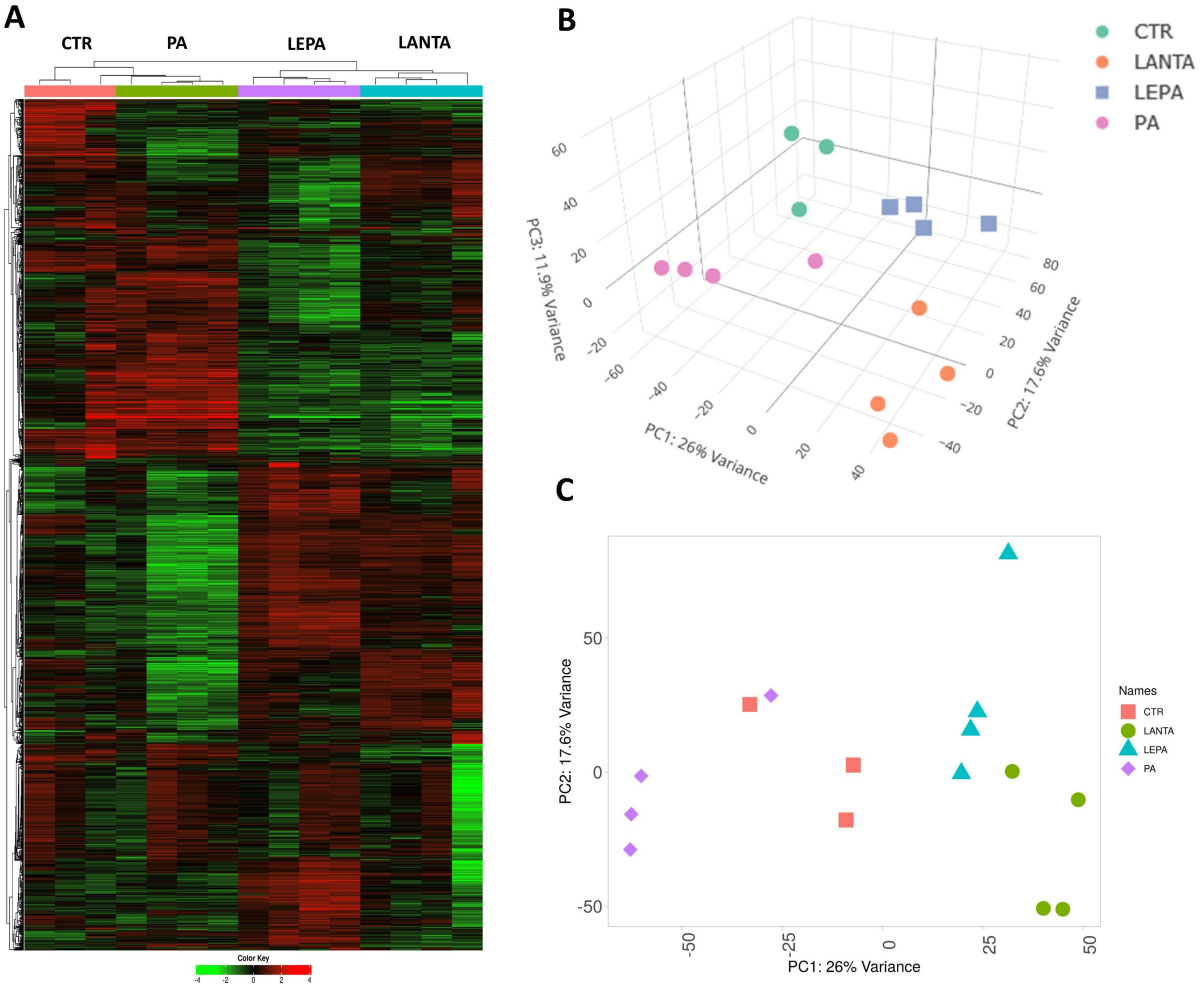
Differentiation of gene expression profiles following different treatments. **(A)** Hierarchically clustered heatmap of gene and sample expression profiles. **(B)** Principal component analysis 3D visualization. **(C)** Principal component analysis 2D visualization.

All filtered reads were used to visualize the differentiation of expression profiles via PCA. Furthermore, a subset of the 2,000 most variable genes was used to prepare a heatmap with hierarchical clustering of genes and sample expression profiles (Figure 2). The PCA revealed visible clusters of samples from different treatment groups (with minor overlaps). Differences in expression profiles among groups were much more apparent in hierarchically clustered heatmaps. Two significant clades were identified for control/PA- and LEPA/LANTA-treated cells, with visible internal differentiation of samples belonging to separate treatment groups. The 2,000 most variable genes were clustered into numerous clades, including large subsets of predominantly up- or downregulated genes in separate treatment groups (Figure 2).

### 3.5 Changes in the neural cell transcriptome following different treatments

**Figure 3:**
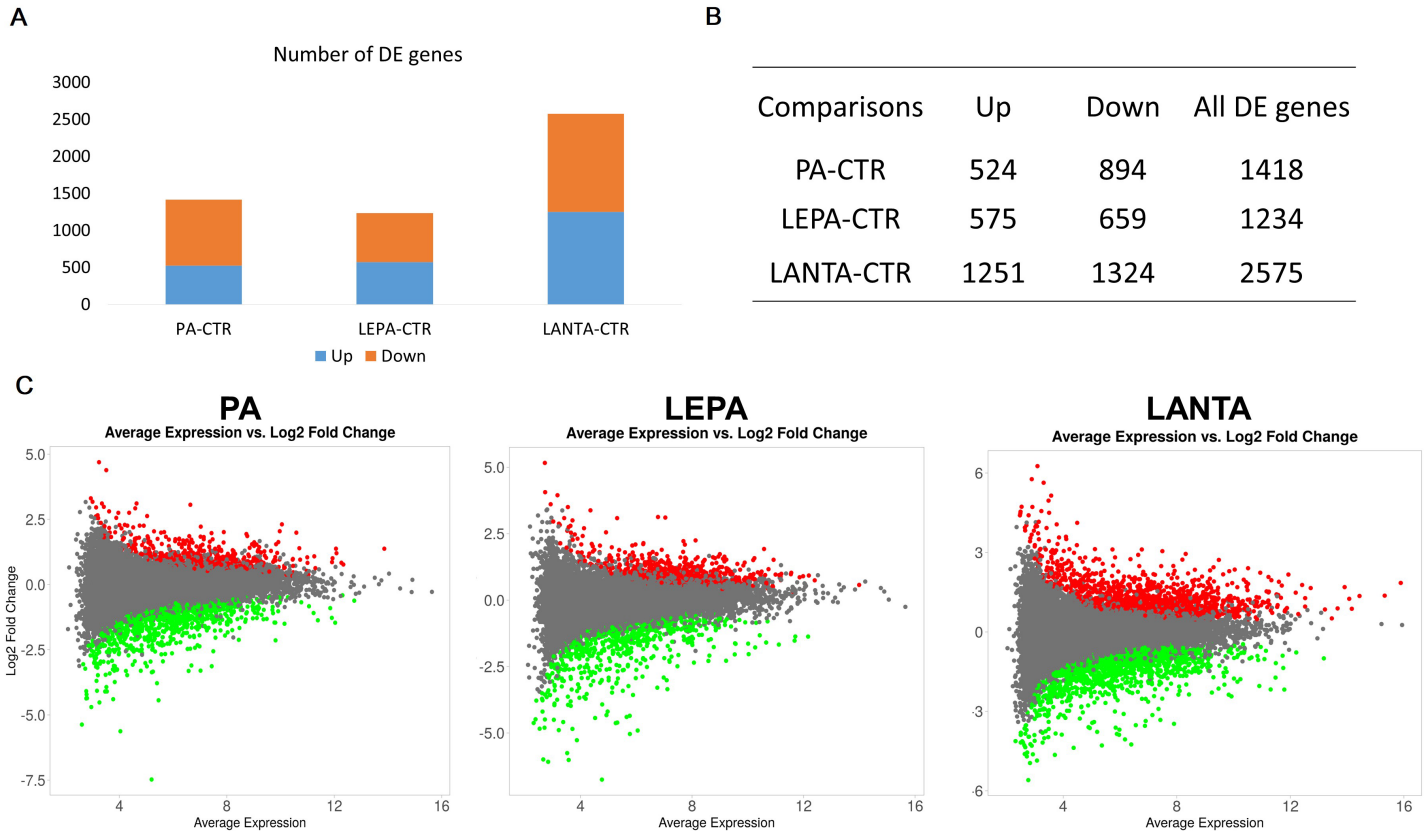
Statistics of genes differentially regulated by various treatments in control untreated cells. **(A)** and **(B)** Numbers and proportions of up-and downregulated genes. **(C)** MA plots of differential gene expression in different treatment groups.

Initially, the transcriptomes of treated cells were compared with those of control cells to capture the basic gene expression alterations caused by each agent. LANTA treatment altered the greatest number of genes (2,575 genes; FDR < 0.05), and LEPA treatment altered the fewest genes (1,234 genes; FDR < 0.05; Figure 3; Supplementary Table S2). In both of those treatments, the numbers of up- and downregulated genes were similar; however, PA treatment caused a predominant downregulation of gene expression (63% of altered genes). Among the differentially expressed genes, 194 were altered in all treated cells (shared genes). The greatest number of altered genes shared by at least two treatments was found in LEPA- and LANTA-treated cells (*n* = 539; Supplementary Table S3).

**Figure 4:**
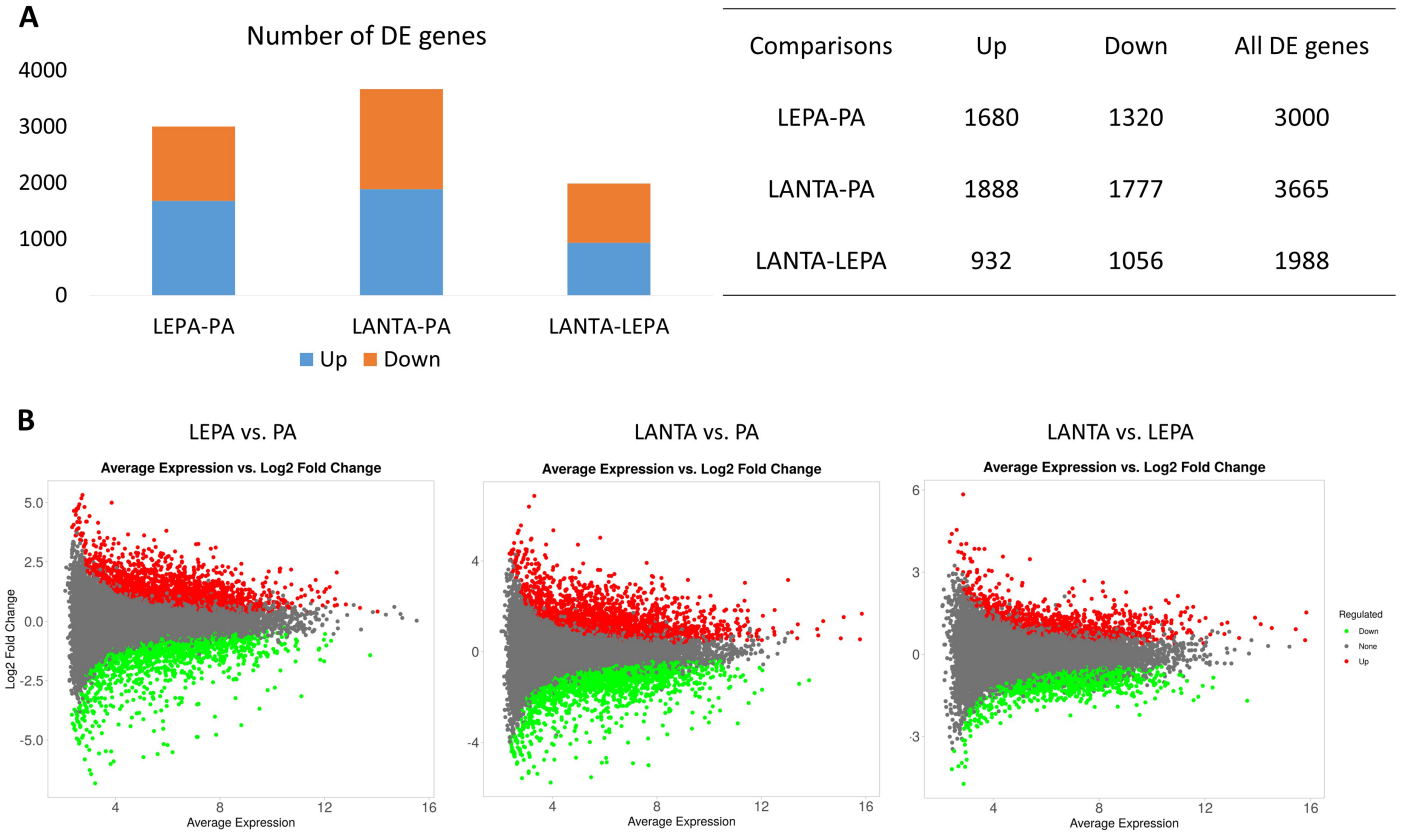
Statistics of genes differentially regulated by various treatments in control untreated cells. **(A,B)** Numbers and proportions of up- and downregulated genes. **(C)** MA plots of differential gene expression in different treatment groups.

A comparison of the transcriptomes of cells treated with LEPA and LANTA revealed 1,988 differentially regulated genes (FDR < 0.05), 1,056 (53%) of which were upregulated by the application of LANTA (Supplementary Table S2). When the transcriptomes of cells from both treatments were compared with those of PA-treated cells, 3,665 genes with altered expression were observed in LANTA-treated cells, and 3,000 were observed in LEPA-treated cells. A slightly greater proportion of genes were upregulated by the LEPA and LANTA treatments than by the PA treatment (56 and 52%, respectively; Figure 4; Supplementary Table S2).

### 3.6 Effects of PA on the neural cell transcriptome

The application of PA to neural cells resulted in altered expression of 1,418 genes. An analysis of genes enriched in GO BP terms revealed that the upregulated genes were enriched (FDR < 0.05) in several categories related to non-coding (mainly ribosomal) RNA processing and ribosome biogenesis (GO:0006396, GO:0006364, GO:0022613, GO:0042254, GO:0034660, GO:0034470 and others), ER stress (GO:0034976, GO:0035966, GO:0030970, GO:1903513, GO:0030433 and others), and protein folding (mainly aberrant) and degradation through the ERAD pathway (GO:0006457, GO:0035966, GO:0006986, GO:0061077, GO:0051084, GO:0036503 and others). In contrast, the downregulated genes were enriched (FDR < 0.05) in BP categories related to mitotic cell cycle regulation and cell division (GO:1903047, GO:0022402, GO:0051301, GO:0010564, GO:1905818, GO:0000070, GO:0140014, GO:0010965, and GO:0051128), metabolic processes or responses to lipids (GO:0006629, GO:0033993) and programmed cell death (GO:0012501, GO:0008219; Figure 5; Supplementary Table S4).

**Figure 5:**
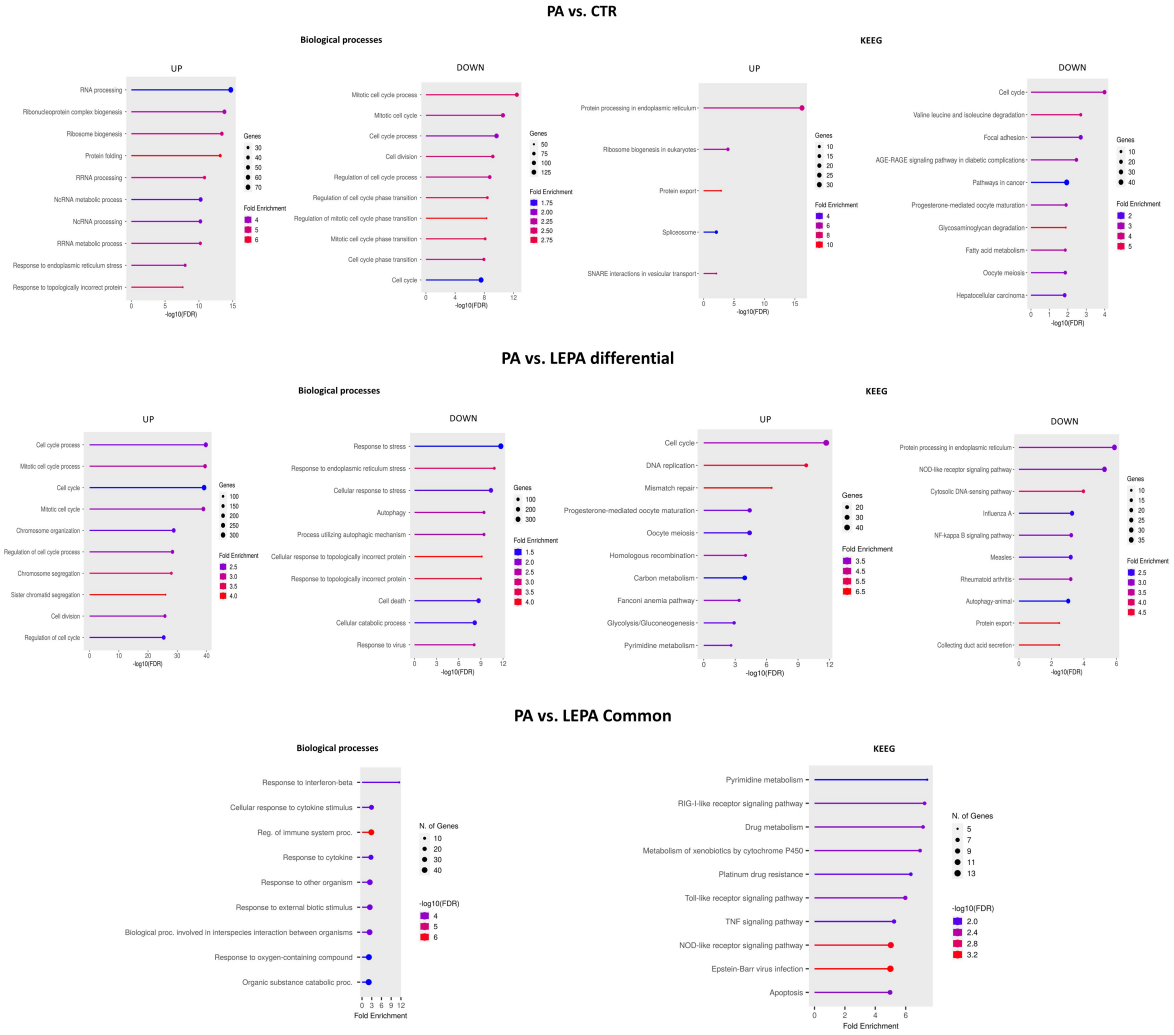
Top 10 biological processes and KEGG pathways enriched with genes altered by palmitic acid (PA) treatment compared with the control and high leptin concentration (LEPA) treatments. The bottom graph presents processes altered by LEPA compared with PA treatment for genes that are commonly differentially regulated in both treatments.

Similar functions of differentially expressed gene were revealed when overrepresentation tests were performed for KEGG pathway categories. The genes upregulated by PA were enriched (FDR < 0.05) in several pathways, e.g., protein processing in endoplasmic reticulum (mmu04141), ribosome biogenesis in eukaryotes (mmu03008), and spliceosome functioning (mmu03040). In contrast, the downregulated genes were associated with cell cycle regulation (mmu04110), focal adhesion (mmu04510), AGE-RAGE signaling pathway in diabetic complications (mmu04933), glycosaminoglycan degradation (mmu00531), fatty acid metabolism and degradation (mmu01212, mmu00071) and several others (Figure 5; Supplementary Table S4).

### 3.7 Comparison of the effects of PA and LEPA treatments on gene expression in neural cells

The effects of PA on gene expression in mouse neural cells were also compared with those of LEPA. In this comparison, 271 genes were commonly dysregulated (Supplementary Table S5), and 3,000 were differentially expressed (Supplementary Table S2). The shared genes were enriched (FDR < 0.05) in GO term categories such as immune system response (e.g., GO:0032727, GO:0035458, GO:0035456, GO:0071345, and GO:0002682), catabolism (GO:0009894, GO:0009057, and GO:1901575) and apoptosis (e.g., GO:0043067, GO:0042981, GO:0010941, and GO:0012501). The overrepresented KEGG pathways included various metabolic pathways (e.g., mmu00980, mmu00240, and mmu01100), immune system-related pathways, e.g., NOD-line, RIG-I-like and Toll-like receptors (mmu05169, mmu04621, mmu04622, mmu05164, mmu04657, and others), and other pathways (Figure 5; Supplementary Table S5).

Within the group of genes differentially regulated between cells treated with PA and those treated with LEPA, genes upregulated by LEPA were associated predominantly with cell cycle regulation/progression and mitotic cell division (GO:0022402, GO:1903047, GO:0010564, GO:0007059, GO:0140014, GO:0044772, and others), whereas downregulated genes were associated mainly with response to stress (also in the ER) (e.g., GO:0006950, GO:0034976, and GO:0033554); cellular response to topologically incorrect or unfolded protein (GO:0061919, GO:0035967, and GO:0006986); catabolic processes (GO:0044248, GO:0009056); cell death, apoptosis and its regulation (GO:0006915, GO:0008219, GO:0012501, and GO:0010941); and immune system response (GO:0009615, GO:0051607, GO:0045071, GO:0016032, and GO:0045087; Figure 5; Supplementary Table S5).

The KEGG pathways associated with genes upregulated by LEPA treatment included cell cycle regulation (mmu04110), DNA replication and repair (mmu03030, mmu03430, mmu03440), glycolysis/gluconeogenesis (mmu00010), focal adhesion (mmu04510) and other pathways. The downregulated genes were associated with a variety of pathways, of which the most interesting ones were protein processing in endoplasmic reticulum (mmu04141) and immune system-related pathways (mmu04621, mmu05164, mmu04064, and mmu04622; Figure 5; Supplementary Table S5).

### 3.8 Effect of LEPA treatment on the neural cell transcriptome

Compared with that in control untreated cells, LEPA altered the expression of 1,234 genes, of which almost equal numbers of genes were upregulated and downregulated, with a minor predominance of downregulated genes (Figure 3). Overrepresentation analysis performed for GO BP terms revealed that the upregulated genes enriched (FDR < 0.05) terms associated with RNA processing (mainly noncoding RNA) and metabolism, which is presumably related to ribosome biogenesis (GO:0006396, GO:0022613, GO:0042254, GO:0016072, GO:0034660, GO:0034470, and GO:0016070); mRNA processing (GO:0008380, GO:0006397, GO:0016071, GO:0016070, and GO:0000375); DNA metabolism, replication and repair (GO:0006259, GO:0006260, GO:0051276, GO:0006261, GO:0006281, GO:0006270 and others); and cell cycle regulation (GO:0007049, GO:1903047). The downregulated genes in LEPA-treated cells were enriched BP terms such as immune system response (GO:0002376, GO:0006952, GO:0006955, GO:0045087, GO:0051607, and others), response against viruses (GO:0019079, GO:0045071, and GO:0045071) and external biotic stimuli (GO:0009607, GO:0043207, and GO:0051707; Figure 6; Supplementary Table S6).

**Figure 6:**
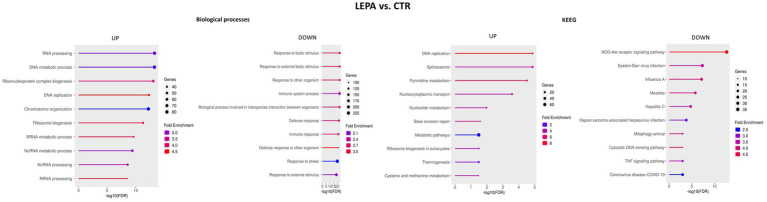
Top 10 biological processes and KEGG pathways enriched with genes altered by high leptin concentration (LEPA) treatment compared with the control.

The KEGG pathways enriched by genes upregulated by LEPA treatment were related to the BP terms and included DNA replication and repair (mmu03030, mmu03410), RNA processing (mmu03040, mmu03018) and ribosome biogenesis (mmu03008), among others. The downregulated genes enriched in KEGG pathways were associated mostly with the immune system response, mainly against viruses (mmu05164, mmu05171, mmu04657, mmu05167) and apoptosis (mmu04210; Figure 6; Supplementary Table S6).

### 3.9 Effect of LANTA treatment on the neural cell transcriptome

LANTA treatment altered the expression of 2,575 genes, almost equal numbers of which were up- and downregulated. The functional analysis of genes upregulated by this treatment revealed enrichment (FDR < 0.05) in BP terms responsible for RNA processing (mainly noncoding) and ribosome biogenesis (GO:0006396, GO:0022613, GO:0034470, and GO:0006364); DNA metabolism, repair and replication (GO:0006259, GO:0006260, GO:0006261, and GO:0006974); mRNA processing (GO:0006397 and GO:0000398); cell cycle regulation (GO:0007049 and GO:1903047); and cellular respiration (GO:0022904, GO:0045333, and GO:0015986). The BP terms in which the downregulated genes were enriched were related to the response to stress by organic stimuli and organic substances (GO:0006950, GO:0070887, and GO:0010033), catabolic processes (GO:0009056, GO:0044248, and GO:0009894), immune system responses (mainly to viruses) (GO:0051607, GO:0002376, GO:0009615, GO:0098542, and GO:0006955), and apoptosis (GO:0008219, GO:0006915, GO:0010941, and GO:0012501; Figure 7; Supplementary Table S7).

**Figure 7:**
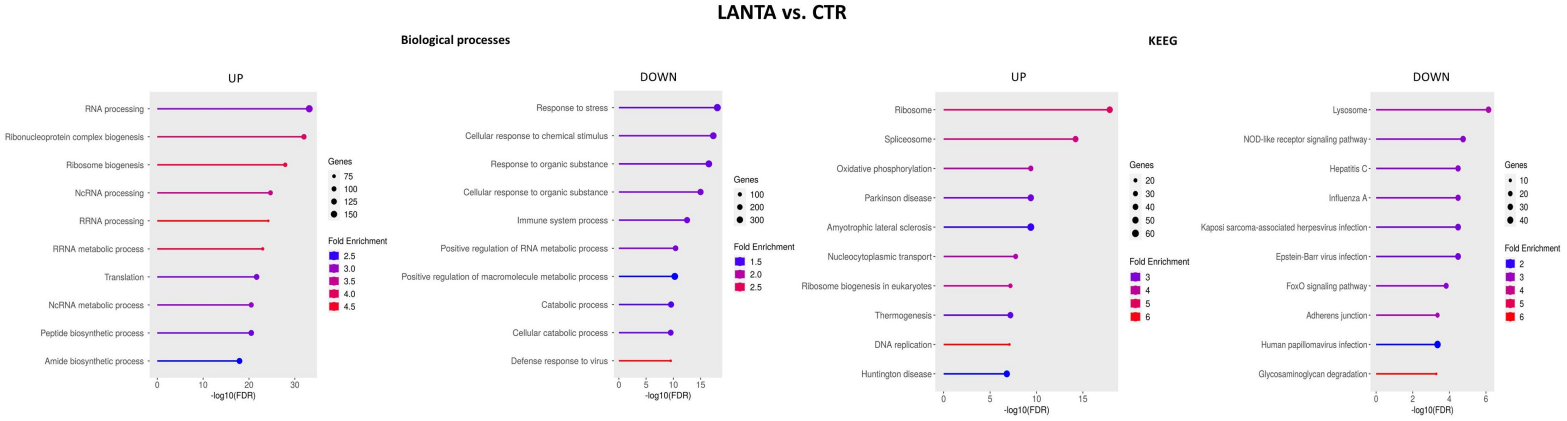
Top 10 biological processes and KEGG pathways enriched with genes altered by leptin receptor antagonist (LANTA) treatment compared with the control.

KEGG pathway analysis yielded similar results, showing that the upregulated genes were involved in ribosome biogenesis (mmu03010, mmu03008), DNA replication and repair (mmu03030, mmu03410), the cell cycle (mmu04110), and RNA splicing (mmu03040). The genes downregulated by LANTA were enriched mainly in pathways related to viral infection (mmu05164, mmu05160, mmu05167, and mmu05165) and immune system processes (mmu04657, mmu04064, and mmu04621; Figure 7; Supplementary Table S7).

### 3.10 Comparison of the effects of LEPA and LANTA on gene expression

Next, the effects of LEPA and LANTA on the neural cell transcriptome were compared. Compared with the control, 733 genes were commonly altered in both treatments, and 1,988 genes were differentially expressed. The shared genes were enriched in BP terms such as immune system response to viruses (GO:0032728, GO:0045071, GO:0035456, GO:0048525, GO:0019079, GO:0140546, and others), metabolism (GO:0006518, GO:0031329, GO:0009894, GO:0043603, and others), DNA damage response and repair (GO:0006974, GO:0006259) and apoptosis (GO:0006915, GO:0042981, GO:0043067, and GO:0010941). The enriched KEGG pathways included those associated with various immune system responses (mmu05340, mmu04621, mmu05169, mmu04657, mmu04668, mmu05160), DNA replication and repair (mmu03030, mmu03410), apoptosis (mmu04210) and oxidative phosphorylation (mmu00190; Supplementary Table S8).

Overrepresentation in BP terms was also analyzed for the genes that were differentially expressed between cells treated with LEPA and those treated with LANTA. The genes upregulated by LANTA were enriched in processes associated with RNA processing (mainly noncoding) and ribosome biogenesis (GO:0006396, GO:0022613, GO:0042254, GO:0006364, GO:0034470, and GO:0008380), translation (GO:0002181, GO:0006412, and GO:0043043), and cellular respiration/ATP synthesis (GO:0006119, GO:0009060, GO:0042776, GO:0010257, and GO:0022900). Most genes downregulated by LANTA treatment were associated with metabolic processes, including those related to RNA metabolism, nitrogen compound biosynthesis, and macromolecule biosynthesis (GO:0044271, GO:0016070, GO:0009891, GO:0009059, and GO:0031328). Other downregulated genes were associated with transcription, RNA splicing and translation (GO:0008380, GO:0010468, GO:0097659, GO:0032774, and GO:0006366; Figure 8; Supplementary Table S8).

**Figure 8:**
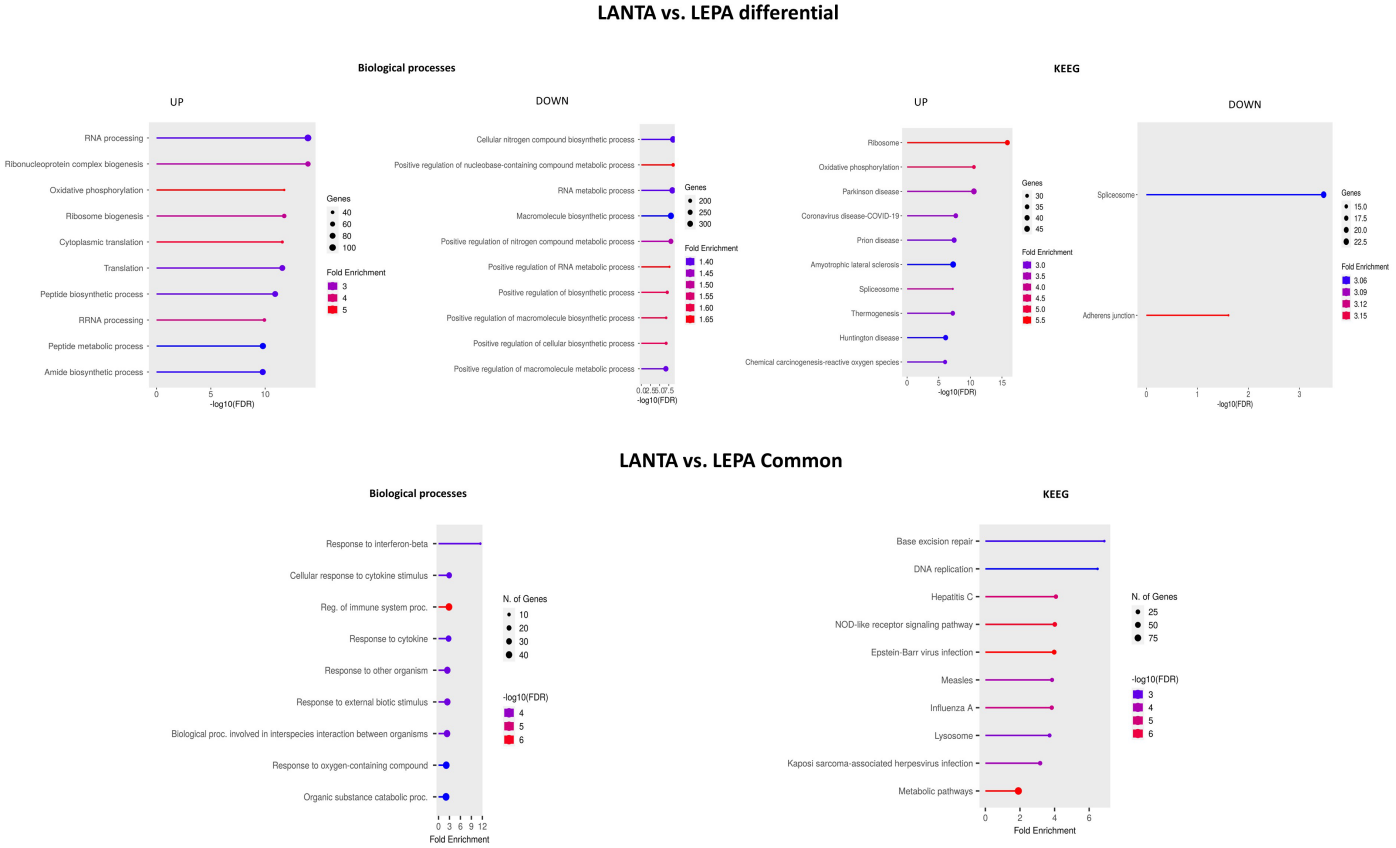
Top 10 biological processes and KEGG pathways enriched with genes altered by leptin receptor antagonist (LANTA) and high leptin concentration (LEPA) treatment.The graphs present processes altered by LANTA compared with LEPA treatment for genes that are commonly differentially regulated in both treatments.

KEGG pathways associated with differentially regulated genes between LANTA- and LEPA-treated cells were identified. Upregulated genes were associated with ribosome functioning (mmu03010), oxidative phosphorylation (mmu00190), cholesterol metabolism (mmu04979) and several disease phenotypes, including neurodegenerative and diabetic or fatty liver diseases (mmu05016, mmu05010, mmu05415, mmu04932, mmu05022). In contrast, genes downregulated by LANTA were connected with only two pathways, namely, spliceosome function (mmu03040) and adherens junction function (mmu04520; Figure 8; Supplementary Table S8).

### 3.11 qPCR validation results

The RNA-Seq validation through qPCR analysis demonstrated strong correlations between the expression levels measured via both methods. Specifically, the correlation coefficient was r = 0.81 (*p* < 0.0013) for *Mapk3*, r = 0.79 (*p* < 0.0021) for *Stat3*, and r = 0.73 (*p* < 0.007) for *Stat2*. These strong correlations indicate the reliability of the RNA-Seq data (Supplementary Table S13).

### 3.12 Differential expression of immune-related genes in response to modulated leptin signaling

**Figure 9:**
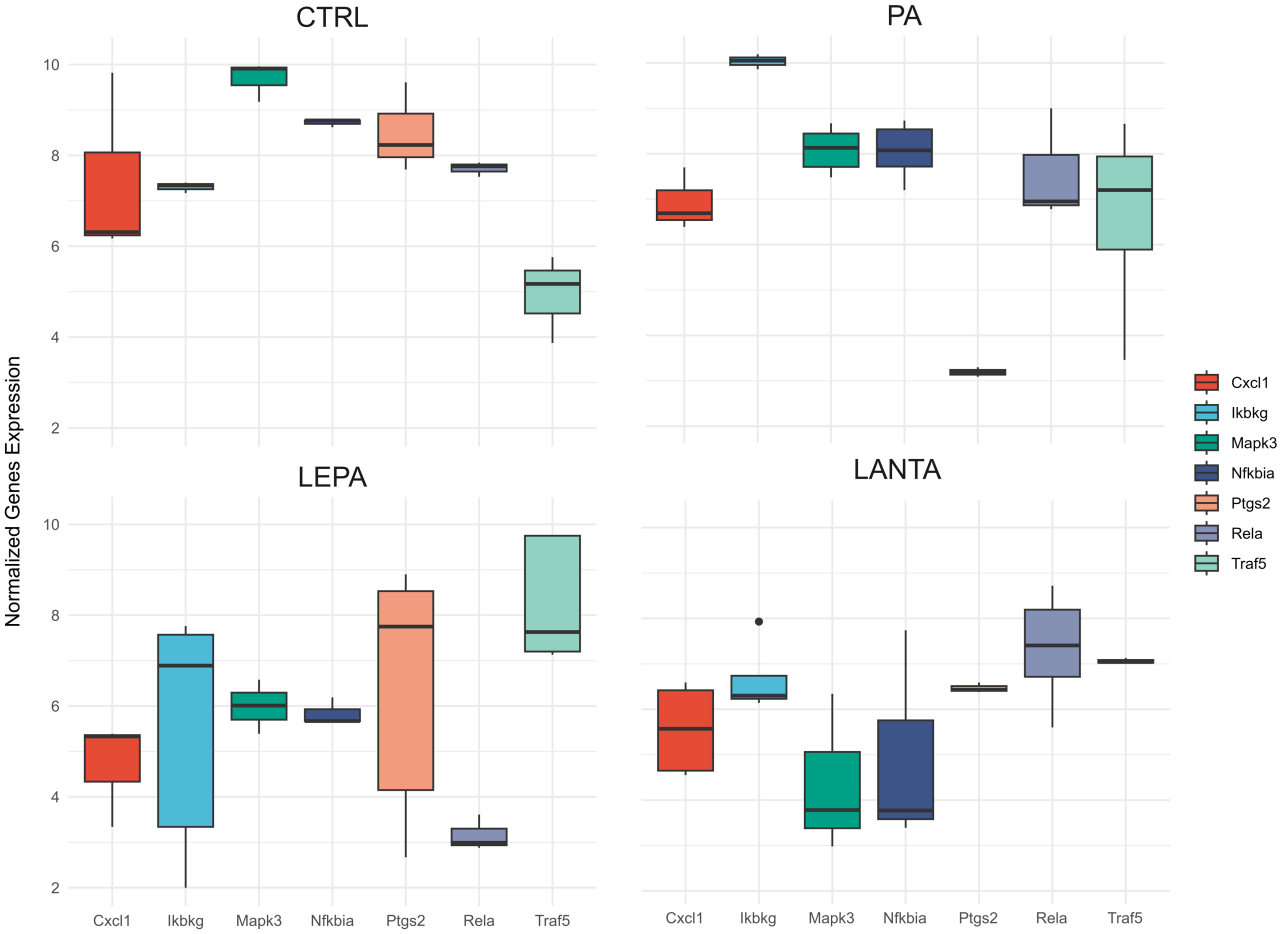
Normalized gene expression of key inflammatory and immune-related genes in hypothalamic neurons following PA, LEPA, and LANTA treatments. The data represent normalized gene counts for genes with significantly altered expression in pairwise comparisons among treated and control groups, determined using the Wald test (with FDR correction). Statistically significant genes, identified in at least one comparison, are presented. Data for all pairwise comparisons have been moved to Supplementary Table S2, specifically to the sheet titled “*Selected Genes*” for clarity.

The analysis of the expression of six genes (*Ikbkg*, *Nfkbia, Rela*, *Mapk3*, *Ptgs2*, and *Cxcl1*), key players in immune pathways, including C-type lectin receptor (Supplementary Table S9), and IL-17 signaling (Supplementary Table S10), NF-kappa B (Supplementary Table S11), NOD (Supplementary Table S12), revealed several significant differences (Figure 9). Compared with CTR, *Ikbkg* was significantly downregulated by PA (log2FC = −0.61, *p* < 0.05), LEPA (log2FC = −1.63, *p* < 0.001), and LANTA (log2FC = −0.90, *p* < 0.001), with the greatest reduction in the LEPA group. *Ikbkg* was also downregulated by LEPA compared with PA (log2FC = −1.02, *p* < 0.001) but upregulated compared with LANTA (log2FC = 0.73, *p* < 0.05). *Nfkbia* was significantly downregulated by LEPA (log2FC = −1.12, *p* < 0.01) and LANTA (log2FC = −1.40, *p* < 0.001) compared with the CTR group, with the greatest reduction in the LANTA group. *Nfkbia* was also significantly downregulated by LEPA (log2FC = −0.88, *p* < 0.01) and LANTA (log2FC = −1.16, *p* < 0.001) compared with PA. *Rela* was significantly downregulated by LANTA compared with CTR (log2FC = −1.19, *p* < 0.001), PA (log2FC = −1.17, *p* < 0.001), and LEPA (log2FC = −0.84, *p* < 0.01). Compared with CTR, *Mapk3* was significantly downregulated by PA (log2FC = −1.30, *p* < 0.001) and LANTA (log2FC = −1.49, *p* < 0.001), with the greatest reduction in the LANTA group. *Mapk3* was upregulated by LEPA compared with PA (log2FC = 0.93, *p* < 0.01) but downregulated compared with LANTA (log2FC = −1.12, *p* < 0.01). *Ptgs2* was significantly downregulated by LEPA (log2FC = −2.91, *p* < 0.001) and LANTA (log2FC = −1.51, *p* < 0.01) compared with CTR, with the greatest reduction in the LEPA group. *Ptgs2* was also significantly downregulated by both LEPA (log2FC = −4.01, *p* < 0.001) and LANTA (log2FC = −2.61, *p* < 0.001) compared with PA and upregulated by LANTA compared with LEPA (log2FC = 1.41, *p* < 0.01). *Cxcl1* was significantly downregulated by LEPA (log2FC = −6.76, *p* < 0.001) and LANTA (log2FC = −3.69, *p* < 0.001) compared with CTR, with the greatest reduction in the LEPA group. *Cxcl1* was significantly downregulated by LEPA (log2FC = −5.72, *p* < 0.001) and LANTA (log2FC = −2.65, *p* < 0.01) compared with PA and upregulated by LANTA compared with LEPA (log2FC = 3.06, *p* < 0.01).

## 4 Discussion

In this study, we utilized the mHypoA-2/12 cell line, a murine hypothalamic model, to explore how the modulation of leptin sensitivity through treatments such as PA, LEPA, and LANTAs alters critical signaling pathways. This cell line expresses the long form of the leptin receptor (LeptRb), making it highly suitable for studying leptin signaling and its disruption under conditions of metabolic stress. Importantly, mHypoA-2/12 cells are NPY/AgRP positive and POMC negative, which reflects their role in energy homeostasis and provides an appropriate model for understanding the molecular mechanisms of leptin resistance in AgRP neurons. Additionally, the expression of several critical receptors (melanocortin 3 receptor (MC3R), insulin receptor (IR), ghrelin receptor (GHSR), and glucagon-like peptide receptor type 1 and 2 (GLP-1R, GLP-2R)) further enhances the utility of these cells in investigating the hypothalamic regulation of metabolism (Dalvi et al., 2011, 2021).

Leptin signaling disruption in hypothalamic neurons plays a critical role in the pathogenesis of leptin resistance, which is a significant contributor to obesity and metabolic disorders. Among the various proposed mechanisms, a growing body of evidence supports the view that leptin resistance, which is mainly induced by overnutrition, is intricately linked to a spectrum of cellular stress responses, including ER stress, oxidative stress, and chronic inflammation (de Git and Adan, 2015; Szczesna and Zieba, 2015; Park et al., 2022). Understanding these mechanisms is crucial for developing effective strategies to combat obesity and its associated disorders, emphasizing the urgency of our research. Our GO enrichment analysis revealed interesting differences in the regulation of genes between cells treated with PA and those treated with LEPA. In particular, PA treatment, which models the effects of an HFD, induced substantial stress responses, as reflected by the upregulation of genes associated with ER stress and protein misfolding, increased caspase-3/7 activity and decreased cell viability. These findings support the hypothesis that PA exacerbates leptin resistance by inducing cellular stress and apoptosis in hypothalamic neurons. This result is consistent with the literature that links HFDs to TLR4 activation and subsequent hypothalamic inflammation, contributing to leptin resistance (Chen et al., 2024). In contrast, genes upregulated by LEPA were predominantly involved in cell cycle regulation and mitotic cell division, suggesting a role for LEPA in promoting cellular proliferation and maintenance. These opposing gene expression patterns between PA and LEPA highlight their distinct impacts on cellular homeostasis. Understanding this dynamic interaction might provide deeper insights into how leptin signaling and cellular responses are modulated, particularly in the context of metabolic disorders such as obesity (Zhou and Rui, 2013; Campolim et al., 2020; Horiuchi et al., 2021). Furthermore, the modulation of leptin sensitivity resulted in significant changes in multiple signaling pathways, with a general downregulation observed across several key inflammatory pathways. Notably, three genes, *Nfkbia, Ikbkg*, and *Rela,* were consistently involved in all four major pathways that were affected: the NOD-like receptor (*p* < 0.0000177), C-type lectin receptor (*p* < 0.00075), NF-kappa B (*p* < 0.00214), and IL-17 signaling (*p* < 0.00767) pathways (Figure 9; Supplementary Tables S9–S12). These genes are known to be critical mediators of immune and inflammatory responses (Liu et al., 2017), particularly through the regulation of NF-kappa B signaling. The description of the role of each of these genes is intentionally limited for clarity. *Nfkbia* encodes the *IκBα* protein, which inhibits NF-kappa B by sequestering it in the cytoplasm. *Ikbkg* (NEMO) is essential for activating NF-kappa B, whereas *Rela* (p65) is a core component of the NF-kappa B complex that mediates transcriptional responses to stress and inflammation (Chen et al., 2024). In addition to these three genes, *Mapk3, Ptgs2*, and *Cxcl1*, which are involved in some but not all of the same pathways, were also identified. MAPK3 (ERK1) plays a role in cellular stress responses and is involved in the NOD-like receptor, C-type lectin receptor, and IL-17 signaling pathways but not in the NF-kappa B pathway (Lee et al., 2009). *Ptgs2* (COX-2), which drives proinflammatory prostaglandin production, is involved in both the C-type lectin receptor pathway and the NF-kappa B signaling pathway (Wang et al., 2012). *Cxcl1*, a chemokine responsible for immune cell recruitment, is part of the NOD-like receptor, NF-kappa B, and IL-17 signaling pathways (Zhao et al., 2010). With PA application, *Mapk3* is significantly downregulated, reflecting the inhibition of the MAPK/ERK pathway. This reduction in ERK1 activity limits the ability of cells to respond to ER stress and protein misfolding, both of which were highlighted by the GO analysis. Since ERK1 typically supports cell survival and repair, its suppression under PA likely exacerbates ER stress, leading to increased apoptosis and reduced cell viability. These findings are consistent with the broader GO analysis, which indicated significant cellular dysfunction in response to PA treatment. In parallel, *Ptgs2* (COX-2), a key enzyme responsible for the synthesis of proinflammatory prostaglandins, has the opposite regulatory pattern. The significant upregulation of *Ptgs2* with PA application intensifies inflammatory signaling, particularly through the NF-kappa B and C-type lectin receptor pathways, driving the proinflammatory environment characteristic of HFD exposure. This increase in *Ptgs2* reflects the enhanced inflammatory state caused by PA, which contributes to cellular damage and stress. Conversely, with both LEPA and LANTA treatments, *Ptgs2* is downregulated, indicating the suppression of COX-2-mediated inflammation. This downregulation indicates a shift toward a less inflammatory state, likely reflecting reduced prostaglandin synthesis and a broader attenuation of NF-kappa B-driven inflammatory pathways. Additionally, *Cxcl1* exhibited significant downregulation (*p* < 2.52E-10) with LEPA application, indicating reduced immune recruitment and inflammatory signaling. CXCL1 plays a crucial role in mediating neutrophil recruitment during inflammation, particularly under acute leptin exposure, where inflammation is typically increased (Souza-Almeida et al., 2018). However, inflammatory pathways can become dysregulated or suppressed during chronic leptin exposure, as observed in leptin resistance. The downregulation of *Cxcl1* under LEPA likely reflects an adaptive mechanism aimed at reducing chronic inflammation in hypothalamic neurons. This finding is consistent with the results of the GO analysis, which revealed that LEPA promoted cell cycle regulation and mitotic division, suggesting a focus on cellular maintenance and repair rather than inflammation.

Unlike POMC neurons, which are often involved in the acute proinflammatory responses mediated by leptin, AgRP neurons may play a more protective role under chronic conditions by suppressing inflammation. Studies have shown that chronic metabolic stress, such as obesity and HFD consumption, leads to chronic hyperactivity of AgRP neurons, which is likely a result of leptin resistance. Although this chronic activation does not always cause long-lasting obesity, this observation highlights the distinct roles of these neurons under chronic conditions, where they may be involved in modulating energy expenditure and metabolism without triggering excessive inflammation (Johnson et al., 2020). Additionally, research on chronic stress has shown that prolonged stress can suppress the activity of AgRP neurons, reducing their contribution to harmful outcomes such as inflammation and stress-related neuronal damage. This suppression suggests a mechanism by which AgRP neurons protect against chronic inflammatory responses during long-term metabolic stress, such as leptin resistance (Fang et al., 2021). Thus, the downregulation of inflammatory pathways, such as the NF-kappa B and IL-17 pathways, observed in our study may be a reflection of this protective mechanism in AgRP-positive neurons. This adaptive response could limit the potential damage caused by chronic inflammation in the hypothalamus during conditions such as prolonged HFD exposure and leptin resistance. Interestingly, LANTA treatment involves the superactive mouse leptin antagonist (SMLA), a potent antagonist of leptin activity that competes with the wild-type (WT) hormone to bind to leptin receptors and has a binding efficiency increased more than 60-fold compared with that of WT leptin (Shpilman et al., 2011). In our study, SMLA treatment resulted in a significant downregulation of *Rela* (*p* < 0.0000162), *Ikbkg* (*p* < 0.00082) and *Nfkbia* (*p* < 0.0000218), indicating suppressed NF-kappa B signaling. The partial recovery of *Ikbkg* suggested some restoration of NF-kappa B activity, particularly within the NOD-like receptor and IL-17 signaling pathways, indicating that leptin antagonism with SMLA could partially restore immune responses suppressed by chronic leptin exposure under PA and LEPA conditions. Additionally, SMLA reduced cellular stress and apoptosis while restoring pathways related to cell cycle regulation and DNA repair, indicating its potential therapeutic role in restoring normal immune and metabolic functions in leptin-resistant states. Using SMLA alone provided a focused model of receptor-level leptin resistance, enabling a clearer comparison with the inflammation- and overstimulation-driven mechanisms induced by PA and LEPA. This approach underscores that while distinct mechanisms underlie leptin resistance, their downstream transcriptional responses can exhibit significant overlap, reflecting shared disruptions in key signaling pathways.

The unexpected downregulation of inflammatory pathways under all conditions contrasts with the typical activation of these pathways observed in acute leptin exposure. However, this suppression may represent a protective feedback mechanism that is activated by chronic metabolic stress, which prevents further neuronal damage. Notably, the similar effects of LEPA and LANTA on immune-related gene expression may reflect shared downstream signaling disruptions, such as in NF-κB and MAPK pathways, despite their differing receptor-level mechanisms. Both treatments likely converge on transcriptional regulation driven by compensatory responses to chronic leptin resistance. Studies have shown that chronic inflammation in the hypothalamus can lead to neuronal damage and that downregulating inflammatory signaling may prevent further harm (Horiuchi et al., 2021). Our findings are consistent with research suggesting that chronic ER stress in the hypothalamus can contribute to both metabolic dysfunction and immune suppression, reflecting the complex interplay between these processes (Campolim et al., 2020; Ye et al., 2021).

### 4.1 Limitations

Although this study provides valuable insights into leptin resistance and its associated signaling pathways, a few limitations should be noted. First, the immortalized mHypoA-2/12 cell line was chosen for its ease of culturing and reproducibility, as well as its expression of LepRb, making it a suitable model for investigating leptin signaling. However, this cell line may not fully represent the complexity of hypothalamic neurons *in vivo*. Future studies using primary hypothalamic neurons could complement our findings despite the challenges in culturing these cells. Second, we employed 3’ mRNA-Seq transcriptome analysis, a method that efficiently quantifies gene expression by targeting the 3’ end of mRNA transcripts. While this approach is suitable for high-throughput analysis and accurate gene quantification, it inherently limits the detection of transcript variants and alternative splicing events, as only the 3’ ends of transcripts are captured.

## Data Availability

The datasets presented in this study can be found in online repositories. The names of the repository/repositories and accession number(s) can be found in the article/Supplementary material.

## Footnotes

^1^ https://jasp-stats.org/

## Supplementary material

Supplementary Figure S1 Sequencing reads, filtering, and mapping statistics for 3’ mRNA-Seq data.

Supplementary Figure S2 Differential gene expression analysis between the treatment groups and the control group and among the treatment groups along with normalized gene expression data.

Supplementary Figure S3 Venn diagram analysis of genes differentially expressed under different treatments.

Supplementary Figure S4 Results of overrepresentation tests in BP terms and KEGG categories for genes affected by palmitic acid (PA) treatment.

Supplementary Figure S5 Results of overrepresentation tests in BP terms and KEGG categories for genes affected by palmitic acid (PA) compared with high leptin concentration (LEPA) treatment. Both commonly affected (compared with the control) and differential (between treatments) genes for these treatments are presented.

Supplementary Figure S6 Results of overrepresentation tests in BP terms and KEGG categories for genes affected by high leptin concentration (LEPA) treatment.

Supplementary Figure S7 Results of overrepresentation tests in BP terms and KEGG categories for genes affected by leptin receptor antagonist (LANTA) treatment.

Supplementary Figure S8 Results of overrepresentation tests in BP terms and KEGG categories for genes affected by high leptin concentration (LEPA) compared with leptin receptor antagonist (LANTA) treatment. Both commonly affected (compared with the control) and differential (between treatments) genes for these treatments are presented.

Supplementary Figure S9 Down-regulation of genes in the KEGG C-Type Lectin Receptor Signaling Pathway following LANTA and LEPA treatments in hypothalamic neurons.

Supplementary Figure S10 Down-regulation of genes in the KEGG IL-17 Signaling Pathway following LANTA and LEPA treatments in hypothalamic neurons.

Supplementary Figure S11 Down-regulation of genes in the KEGG NF kappa B Signaling Pathway following LANTA and LEPA treatments in hypothalamic neurons.

Supplementary Figure S12 Down-regulation of genes in the KEGG NOD-like receptor Signaling Pathway following LANTA, LEPA and PA treatments in hypothalamic neurons.

Supplementary Figure S13 qPCR validation of RNA-seq data for Mapk3, Stat3, and Stat2, including assay IDs, amplicon lengths, and Pearson correlation coefficients (r) with statistical significance.
