# Supplementary figures and images for: Impact of modulating leptin sensitivity on the transcriptomic profile of adult-derived hypothalamic mouse neurons
Source: Front Mol Neurosci. 2025 Jan 17;17:1518737. doi: 10.3389/fnmol.2024.1518737 (PMC11800294; doi:10.3389/fnmol.2024.1518737)

Dendritic cell, macrophage, neutrophil, etc

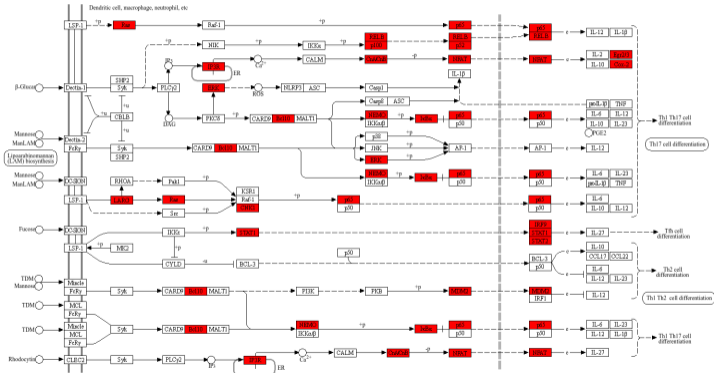

Supplement: Supplementary Figure S9 — Down-regulation of genes in the KEGG C-Type Lectin Receptor Signaling Pathway following LANTA and LEPA treatments in hypothalamic neurons. [file Data_Sheet_9.pdf]

**LANTA**

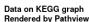

**LEPA**

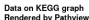

Supplement: Supplementary Figure S10 — Down-regulation of genes in the KEGG IL-17 Signaling Pathway following LANTA and LEPA treatments in hypothalamic neurons. [file Data_Sheet_10.pdf]

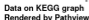

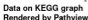

Supplement: Supplementary Figure S11 — Down-regulation of genes in the KEGG NF kappa B Signaling Pathway following LANTA and LEPA treatments in hypothalamic neurons. [file Data_Sheet_11.pdf]

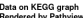

Supplement: Supplementary Figure S12 — Down-regulation of genes in the KEGG NOD-like receptor Signaling Pathway following LANTA, LEPA and PA treatments in hypothalamic neurons. [file Data_Sheet_12.pdf]
